# Classification of Anticipatory Signals for Grasp and Release from Surface Electromyography

**DOI:** 10.3390/s16111782

**Published:** 2016-10-25

**Authors:** Ho Chit Siu, Julie A. Shah, Leia A. Stirling

**Affiliations:** 1 Department of Aeronautics and Astronautics, Massachusetts Institute of Technology, 77 Massachusetts Ave, Cambridge, MA 02139, USA; julie_a_shah@csail.mit.edu (J.A.S.); leia@mit.edu (L.A.S.); 2 Computer Science and Artificial Intelligence Lab, Massachusetts Institute of Technology, 77 Massachusetts Ave, Cambridge, MA 02139, USA; 3 Institute for Medical Engineering and Science, Massachusetts Institute of Technology, 77 Massachusetts Ave, Cambridge, MA 02139, USA

**Keywords:** surface electromyography, Gaussian mixture models, hidden Markov models, machine learning, pattern recognition

## Abstract

Surface electromyography (sEMG) is a technique for recording natural muscle activation signals, which can serve as control inputs for exoskeletons and prosthetic devices. Previous experiments have incorporated these signals using both classical and pattern-recognition control methods in order to actuate such devices. We used the results of an experiment incorporating grasp and release actions with object contact to develop an intent-recognition system based on Gaussian mixture models (GMM) and continuous-emission hidden Markov models (HMM) of sEMG data. We tested this system with data collected from 16 individuals using a forearm band with distributed sEMG sensors. The data contain trials with shifted band alignments to assess robustness to sensor placement. This study evaluated and found that pattern-recognition-based methods could classify transient anticipatory sEMG signals in the presence of shifted sensor placement and object contact. With the best-performing classifier, the effect of label lengths in the training data was also examined. A mean classification accuracy of 75.96% was achieved through a unigram HMM method with five mixture components. Classification accuracy on different sub-movements was found to be limited by the length of the shortest sub-movement, which means that shorter sub-movements within dynamic sequences require larger training sets to be classified correctly. This classification of user intent is a potential control mechanism for a dynamic grasping task involving user contact with external objects and noise. Further work is required to test its performance as part of an exoskeleton controller, which involves contact with actuated external surfaces.

## 1. Introduction

Upper-extremity exoskeletons have demonstrated the potential to assist disabled individuals with the activities of daily living [1], as a part of rehabilitation programs following neurological and/or physical disease or injury [2] and to augment the natural capabilities of healthy individuals [3,4]. Surface electromyography (sEMG) is a feasible method for providing a control signal for device actuation [5,6,7,8] for both exoskeletons and prosthetics. sEMG-based control offers the advantage of an interface that works directly with muscle signals received from natural human motion, providing an intuitive actuation method; an important factor for user acceptance of prosthetic/exoskeleton devices [9].

Some sEMG-based control methods have incorporated proportional or proportional-derivative control using signals received from one or more sEMG sensors [10,11]. However, pattern-recognition-based control has become more common in recent years due to its ability to differentiate between multiple types of limb functions, an ability that classical control methods lack [5,6,7,8,12,13]. Wolf et al. [14,15] and Oskoei and Hu [16] both developed multi-class support vector machine (SVM) methods for classifying static hand and arm gestures. Wolf et al. [14,15] were then able to use their system to control the movements of a mobile robot and a prosthetic hand. These methods were typically able to achieve accuracies of 90%–95%, using just sEMG [16] and both sEMG and IMU data [14].

Gaussian mixture models (GMM) are another method of pattern classification that has been used for sEMG-based teleoperation. Here, Gaussian probability distributions are used to map input signal features to robot motions through the use of training data. When in operation, new input signals cause a selection of a robot response based on the likelihood of the signals belonging to a particular mapping. Fukuda et al. [17] and Artemiadis and Kyriakopoulos [18] both used sEMG and IMU data along with GMMs for teleoperation of a robotic arm based on the in-air movements of a person’s arm. Fukuda [17] reported 70%–98% accuracy, depending on the gesture being identified, and Artemiadis and Kyriakopoulos [18] reported 81%–94% accuracy, depending on the decoding scheme.

An extension of the GMM technique is to use them within the framework of hidden Markov models (HMM). HMMs offer an advantage over many other models because the model inherently considers the stochastic nature of sEMG signals by encoding likely transitions from one intent to another based on common sequences of limb movements, giving greater probability to intent transitions that are likely to follow one another [13,19], such as a “grasp” action following a “reach” action. In this way, the structure of an HMM is particularly suited for classifying sequences over time, more so than classifiers that would consider feature inputs independently from time step to time step (including GMM and SVM). Features have a certain probability of being observed when user intent is in a given state, allowing observables collected over time to be used to determine changes in user intent [19,20]. Depending on the nature of the observables, HMMs may have either discrete or continuous observation spaces. In the discrete case, an emission matrix is used to determine the mapping from an observation to a hidden state probability, but in the continuous case, the emissions are represented by a probability distribution for each hidden state, which is most commonly a Gaussian. The hidden states of both cases are necessarily discrete [19].

Chan and Englehart [13] developed an HMM-based continuous classification system using sEMG signals that recognized six distinct static in-air limb motions (wrist flexion, wrist extension, supination, pronation, hand open and hand close) performed by healthy participants, with an average accuracy of 94.63% when each motion was performed for a duration of 5 s. Marchal-Crespo et al. [21,22] tested motion intent recognition among stroke survivors using features derived from autonomic nervous system (ANS) responses (electrocardiogram, respiration rate, blood pressure and skin conductance) as inputs to their HMM. Despite the physical inability to complete a grasping task with their impaired hands, these participants nonetheless displayed ANS features when they attempted a grasping motion that allowed the HMM to classify their intentions with 72.4% accuracy. Both Chan and Englehart [13] and Marchal-Crespo et al. [22] used continuous HMM formulations for their work.

It should be noted, however, that the predictions made by both Chan and Englehart [13] and Marchal-Crespo et al. [22] relied on a non-causal HMM decoding method where future information was used. In both cases, the posterior probability of being in any state at a given time was calculated by the forwards-backwards algorithm, in which the backwards component is a post hoc method for smoothing out posterior predictions using the part of the observation that is beyond the prediction time point [19]. Chan and Englehart [13] also used a voting technique to further remove spurious predictions using probabilities surrounding a particular point in time in both the past and the future. Although such smoothing is useful and appropriate for post hoc evaluation, it is difficult to evaluate their results in light of an online use case, such as an exoskeleton or prosthetic controller, where delays must be minimized and future information is not available.

While previous papers studied steady-state gesture detection, we extend this work into earlier detection of transient signals for dynamic gesture recognition to enable improved human-exoskeleton fluency. Studies have shown that humans pre-shape their hands when reaching for an object, with visual cues and mental models of the object’s weight and other physical features strongly influencing the associated muscle activity [23]. Prior work by Beckers et al. [24] indicated that anticipatory signals exist in sEMG data for grasp and release actions performed by healthy individuals interacting with objects in their environment, which is consistent with with previous work [25] highlighting anticipatory cortico-cortical inputs to the motor cortex during grasping. However, Beckers et al. [24] also found that the exact placement of the sEMG sensors affected signal output sufficiently, such that even small misalignments (on the order of a centimeter) changed the resulting sEMG features in statistically-significant ways, confirming the variability observed by Gazzoni et al. [26] in sEMG signals detected from the forearm. This variability is of particular importance for the use of sEMG signals as control inputs because expert placement of sensors for a prosthetic or rehabilitative system is currently required for sEMG-based control of such devices. Inter-user physiological variations, such as the amount of hair or fat under a particular sensor, also increase the difficulty of using sEMG-based control [5]. Such changes in the control signal would dramatically affect the results of a classical controller, but an approach based on pattern recognition may be more robust to these variations.

The purpose of this study was to evaluate if pattern-recognition-based methods could classify transient, anticipatory sEMG signals and to characterize the sensitivity of the classifier to parameters required for the method. This evaluation was performed using data from a protocol that included object interaction and variability in sensor placement, which is representative of an operational use case for grasping in a natural environment. In this study, we compared GMMs and HMMs with different classifier parameters and selected a classifier for further characterization. The sensitivity of the best-performing classifier was analyzed for variations on how the training data were labeled and the use of a voting scheme for smoothing. Understanding how pattern-based classifiers are affected by parameter selection is important for extending these methods to dynamic motions in the natural environment.

## 2. Materials and Methods

### 2.1. Data Collection

This study is a secondary analysis of work first reported by Beckers et al. [24], which tested 20 participants. Due to data collection issues, only 16 participants’ data could be used for analysis. Of the 16, there were 10 males and 6 females, and 12 were right-handed. Other subject characteristics are presented in Table 1. All participants were healthy and had no self-reported disabilities on either arm or hand during a pre-study screening. The experiment protocol was approved by the MIT Committee on the Use of Humans as Experimental Subjects, and all participants provided written informed consent. All participants received $15 as compensation.

#### 2.1.1. Task

**Figure 1:**
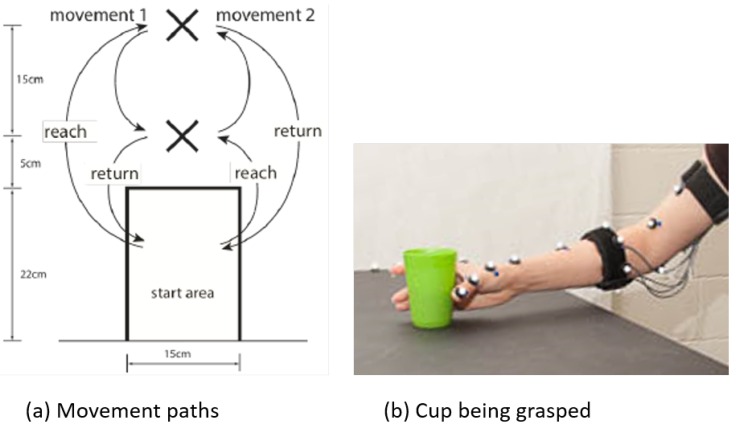
Schematic representation of the task (**a**) and a picture of the cup being grasped, along with the markers and band being used (**b**). Arrows in (**a**) indicate the reach and return for each movement, and “X” symbols mark the object locations. (**a**) reproduced with permission from Beckers et al. [24].

During the task, participants were seated at a table and used their dominant hand to move an object on the table from a predetermined location to a secondary location and back (Figure 1a). Participants were not given specific speed or accuracy objectives and were allowed to complete the task with a self-selected strategy. Each trial consisted of two grasp-and-release sequences (termed “movements”). For the first movement, participants reached forward and grasped the object, moved it approximately 15 cm toward themselves to the secondary location, then released the object and returned their hand to the start area. For the second movement, participants reached forward to the secondary location, grasped the object and then returned it to the first location before releasing the object and returning their hand to the starting area. The objects being transported were a cup (diameter: 6 cm; height: 9 cm; mass: 0.2 kg) and a rod with dimensions and mass similar to a ballpoint pen. These objects were chosen as they are common grasps in activities of daily living that require different grasp strategies. The present secondary analysis examined the use of sEMG signals in the presence of sensor misalignments and uses only the cup trials to minimize additional variability due to grasp strategy. Additional study of grasp strategy in future work is warranted.

#### 2.1.2. sEMG Sensor Band Configurations

**Figure 2:**
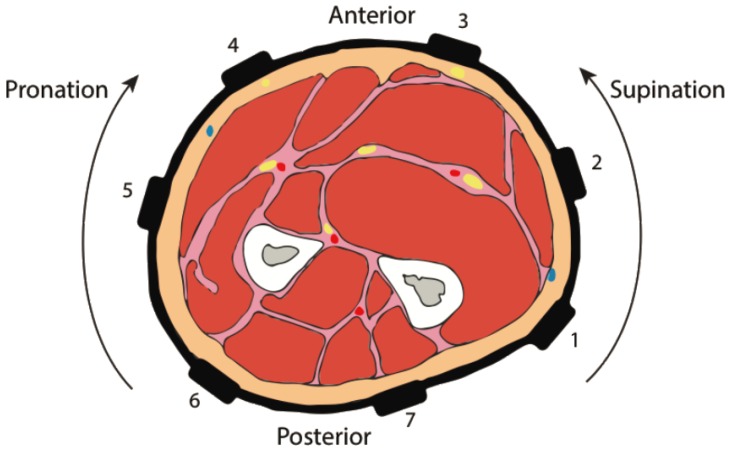
Schematic representation of the sensor band placed in the center configuration around a right forearm cross-section, looking toward the elbow. Figure reproduced with permission from Beckers et al. [24].

Seven sEMG sensors were equally distributed and integrated into a stretchable band placed around the forearm of the participant (Figure 2), such that sensors were not deliberately placed over particular muscles. Data were collected from five configurations of the band. The center configuration was defined with a mark placed on the participant’s arm at 25% the length of the ulna (as measured from the lateral epicondyle) and superficial to the ulna when the participant’s hand was placed on the table. The remaining four configurations were defined as 1 cm up (proximal), down (distal), clockwise (supination) and counterclockwise (pronation) with respect to the center configuration.

#### 2.1.3. Procedure

Participants performed dexterity and hand strength tests with both hands, followed by reaching motions using each of the five sensor band configurations, with 20 trials performed per configuration (10 trials per object). The sensor band position was shifted after each configuration block, with the order of the five configurations counterbalanced across participants.

#### 2.1.4. Data Acquisition

Kinematic data were recorded using a VICON Bonita system (VICON Motion Systems Ltd., Oxford, UK) at a rate of 120 Hz. Fifteen reflective markers were placed on the participants’ shoulders and clavicle, as well as on the elbow, wrist, metacarpals, index finger and thumb of the dominant arm and hand. sEMG data were collected with a DELSYS Bagnoli system (DELSYS Inc., Natick, MA, USA) at 960 Hz from seven double-differential, dry-contact sEMG sensors integrated into a fabric band. The raw data were processed with a 6th-order Butterworth filter (10–450 Hz), followed by a 60-Hz notch filter to remove any noise from surrounding electrical equipment. Data were then mean-subtracted and rectified.

### 2.2. Data Pre-Processing

Voltages were recorded from the seven sEMG sensors, along with estimated times for the start of grasp and release actions as determined by calculations on marker positions and velocities from the motion capture dataset [24]. sEMG data were divided into time epochs: windows of time for which a set of features may be calculated. Epochs were 125 ms in length, with a 100-ms overlap between adjacent epochs. This epoch size was chosen because it was between the 50 and 500 ms epochs used in the literature [15,27,28,29,30] and would allow some processing time if the system was to be used as a controller. Epoch overlap of 80% was also used previously by Wolf et al. [15].

**Figure 3:**
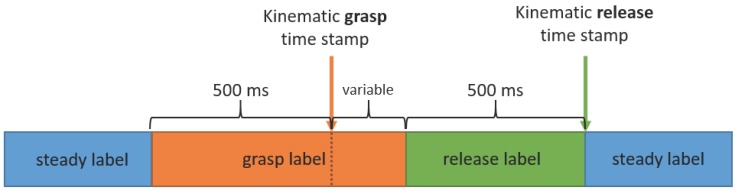
Timeline of relationships between kinematic grasp and release time stamps and labels defined for the GMM/HMM formulation for a single grasp-release sequence.

The epochs occurring within 500 ms before the start of each release action (the kinematic release time stamp) were normally labeled as epochs of release unless a portion of that time crossed over the start time of a grasp action, in which case the labeling of the release action would be shorter than 500 ms (Figure 3). There were relatively few instances of this across all trials (36 out of 1466 total movements, or 2.46%). All epochs occurring between 500 ms before the start of grasp (the kinematic grasp time stamp) and the start of the release labels were then labeled as grasp actions.

All remaining unlabeled epochs were labeled as “steady” to indicate times during which the participant was neither grasping nor releasing an object. The resulting label sequences were considered the kinematically-labeled ground truth intent of the participant. A typical movement sequence was comprised of sets of such labels, transitioning from steady to grasp, to release and back to steady, as shown in Figure 3. We hypothesized that each trial would involve two repetitions of this sequence, as a trial consisted of two movements.

The features extracted from the sEMG time series were integrated absolute value (IAV), waveform length (WFL), variance (VAR), slope sign change (SSC), zero crossings (ZC), Wilson amplitude with a threshold of 5 mV (WAMP), three equally-spaced frequency bins from 10–450 Hz (F1, F2, F3) and three equally-spaced amplitude bins of a size of 0.66 V centered around 0 V (H1, H2, H3) [8,24,31]. Features that were directly mathematically related to these (e.g., root mean square, mean absolute value) were not considered because they did not offer additional information for classification. Correlation testing was done for all feature values across all sensors, and VAR was removed from the feature set due to a high correlation ($R^{2}>0.9$) with IAV across all seven sensors.

### 2.3. GMM and HMM Formulation

Gaussian mixture models with 77 dimensions were made using the feature set (11 features from each of 7 sensors), which were then used as classifiers for epoch-by-epoch predictions. The probability of a set of features *F* and GMM parameters $\theta =\{K,{\left\{\rho^{k},\mu^{k},\Sigma^{k}\right\}}_{k=1}^{K}\}$ is given by:

(1) $$P_{GMM}\left(F|\theta \right)=\sum_{k=1}^{K}\rho^{k}N\left(F|\mu^{k},\Sigma^{k}\right),$$

where *K* is the number of mixture components (Gaussians) and $\rho^{k}$, $\mu^{k}$ and $\Sigma^{k}$ are the mixing proportion, mean and variance of the *k*-th component. A GMM is thus a weighted sum of *K* Gaussian components in some number of dimensions (77 dimensions here). The label choice was found using the maximum likelihood estimate,

(2) $$C_{e}=argmax_{c\in C}P_{GMM}\left(F_{e}|\theta_{c}\right),$$

where the label $C_{e}$ of epoch *e* is the label in C={steady,grasp,release} for which the probability of the observed features $F_{e}$ is greatest. A bigram variation of this method was also used, where 154-dimensional Gaussians were used, corresponding to the current and previous epoch’s feature values [32].

The Gaussian distributions were used in both their unigram and bigram forms as a set of emission probabilities, meaning each state label (hidden state) had a corresponding 77- or 154-dimensional GMM, which gave the likelihood of observing a particular set of features when in a give state. The continuous-emission hidden Markov model was then defined by the following:

(3) $$\Pi =\left[\begin{array}{ccc} \pi_{S} & \pi_{G} & \pi_{R} \end{array}\right]=\left[\begin{array}{ccc} 0.33 & 0.33 & 0.33 \end{array}\right],$$

(4) $$T=\left[\begin{array}{ccc} T_{S,S} & T_{S,G} & T_{S,R}\\ T_{G,S} & T_{G,G} & T_{G,R}\\ T_{R,S} & T_{R,G} & T_{R,R} \end{array}\right],$$

(5) $$E=\left[\begin{array}{ccc} e_{S}\left(o_{i}\right) & e_{G}\left(o_{i}\right) & e_{R}\left(o_{i}\right) \end{array}\right],$$

(6) $$O=\left[\begin{array}{cc} o_{1} & o_{2}\cdots o_{N} \end{array}\right],$$

where the *S*, *G* and *R* subscripts refer to the steady, grasp and release states, respectively. **Π** is the initial probability vector, set to be equal among the three states, even though we know that the initial state is steady, because we wish to test the system under more general conditions, where the user can start in any state. T is the transition probability matrix of transitioning from state *i* to state *j*, developed through counting transitions in the training data and applying Laplace smoothing [32] to avoid zero probabilities. E is the emission probability set of Gaussians; O is the sequence of observations over epochs; and *N* is the total number of epochs. Both unigram and bigram forms of the features were used, for which the Gaussians and observations were of the appropriate dimensionality.

### 2.4. Training and Testing

Only inter-subject training and testing was performed due to high variability in EMG signals between individuals stemming from differences in physiology [8]. Data collected under different band configurations were pooled for processing. Since there were approximately 45 trials per subject (some had fewer due to data quality issues), K-fold cross-validation was performed, with K of 9 or 10, depending on the number of trials available. All training and testing were performed on a Lenovo ThinkPad T440s laptop (Lenovo Group Ltd., Beijing, China) running Windows 10 and MATLAB 2016a.

In the GMM cases, the expectation-maximization algorithm with *K*-means++ initialization [33] was used to fit each set of features associated with a given label to a multivariate Gaussian. For a given set of test data, the probability of a particular set of features belonging to a given label was found by extracting the appropriate value from each label’s distribution function, and the label with the highest probability was used as a predicted label. The same procedure was followed in the HMM cases, except the GMM distributions were now the emission probabilities (Equation (5)), which were used in conjunction with the transition probabilities (Equation (4)) for the prediction.

For the purposes of label prediction in the two HMM cases, the forward algorithm was used rather than the forward-backward algorithm typical for HMM decoding seen in the literature [13,22], even though we have the full sequence of states and features because we are assuming an application where classification is occurring in real time (e.g., for exoskeleton control). The algorithm must then only have information from past and present features (as in the forward case) and not be affected by features later in the sequence (the backward case) [19].

Mixture models with one to fifteen components were tested for unigram and bigram data and both GMM and HMM methods. In all cases, the mixture model regularization value was kept at 0.05. For the selected model, the length of time for which a kinematic release label was applied was also varied between 200 and 600 ms to examine the effect of the label length on these accuracies. Additionally, the effect of changing the epoch size from 50–500 ms (from the default of 125 ms) was also examined.

Finally, we tested the effect of a unanimous voting scheme using only previous information. Here, the classifications made by the maximum likelihood estimate (Equation (2)) were not directly applied to each epoch. Instead, a label was carried over from previous classifications after *n* classifications, until there was unanimous agreement between the present classification and the previous *n* classifications on a different label. This scheme is similar to the majority voting method used by Englehart et al. [34] and Chan and Englehart [13], except rather than using classifications preceding and following the current epoch, we only use preceding epochs, as classifications of future epochs would not be available in an online use case.

### 2.5. Statistical Analysis

In the classifier-selection phase, we analyzed the accuracies with respect to hypotheses related to (1) HMM vs. GMM, (2) bigram vs. unigram and (3) numbers of mixture components. For all three types of tests, the accuracies returned were non-normal, as were residuals of an ANOVA (analysis of variance) on these accuracies, so nonparametric techniques were used. A Kruskal–Wallis test was performed with four groups: unigram-GMM, bigram-GMM, unigram-HMM and bigram-HMM. Post hoc Conover–Inman tests were performed. A separate Kruskal–Wallis test was conducted across the number of components, as well as the corresponding Conover–Inman pairwise tests. For a 5-component HMM unigram case, accuracies returned by varying the length of an epoch from 50–300 ms (with 25-ms shifts between them) were normally distributed. Thus, an ANOVA model was used, along with post hoc Tukey’s tests for pairwise comparisons. Similarly, the number of voting epochs was also varied and returned normally-distributed accuracies, so ANOVA and Tukey’s tests were also used. Statistical tests were performed in SYSTAT 13 (SYSTAT Software Inc, San Jose, CA, USA) and MATLAB 2016a (Mathworks Inc, Natick, MA, USA).

## 3. Results

### 3.1. Classification Accuracy

**Figure 4:**
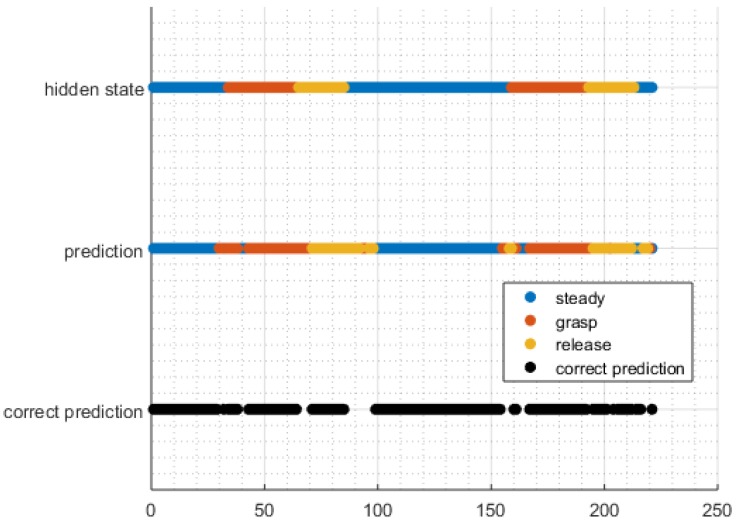
Example of a set of labels over the course of one trial (two grasp and release motions) using the unigram HMM method with five components. Each epoch is 125 ms, with a 100-ms overlap between adjacent epochs.

An example of a set of predictions for a sequence of features is shown in Figure 4. In this case, as in most prediction sequences, we see that misclassifications occur most often during epochs when the user is transitioning from one state to another, and classification is fairly robust when the user has been in a state for a longer period of time.

**Figure 5:**
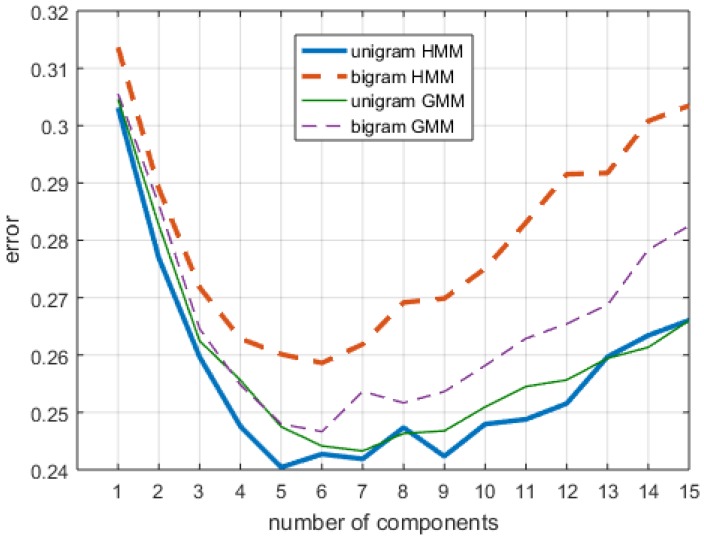
Classification error as a function of the number of mixture components, n-gram differences and classifier type. Standard deviations had a mean of 8.43% and a range of 3.38% across all treatments.

Figure 5 shows the results of the cross-validation of the four classification methods over 755 total trial sequences, each of which was comprised of two grasp-and-release motions. The highest mean accuracy was found by a five-component unigram HMM, which gave an accuracy of 75.96%, with a standard deviation of 8.20%.

**Figure 6:**
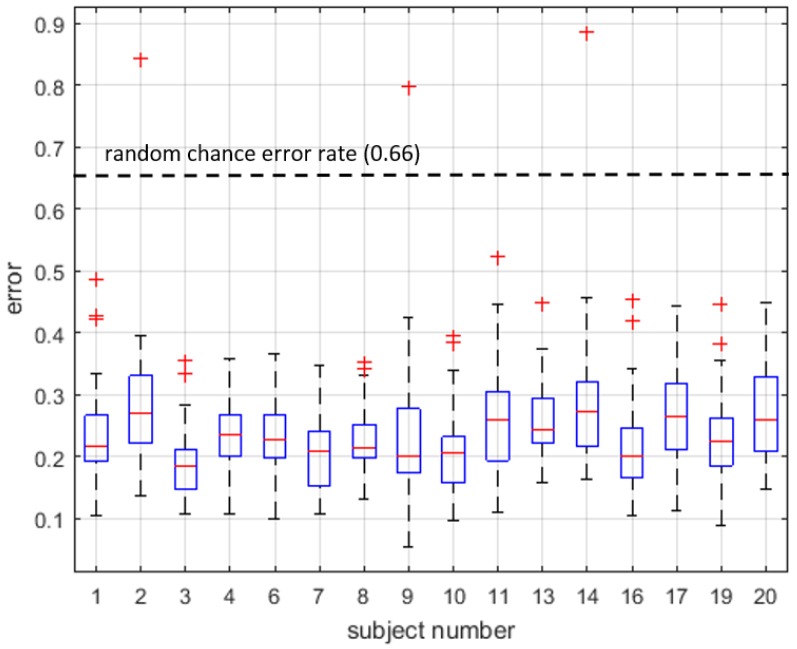
Comparison of classification error by subject for a five-component unigram HMM.

A Kolmogorov–Smirnov test showed that the accuracies were were non-normal (p<0.0001). This may also be seen in Figure 6, which breaks down error rates by subject and where the outliers (red crosses) are all cases with higher errors. A Kruskal–Wallis test found a significant effect of the four groups, consisting of unigram-GMM, bigram-GMM, unigram-HMM and bigram-HMM (H(ν=3)=463.8 and p<0.0001). A post hoc Conover–Inman test for pairwise comparisons found a significant difference for all pairwise comparisons (p<0.0001). A second Kruskal–Wallis test found a significant effect of the number of components, when pooling all data types and algorithms with the same number of components (H(ν=14)=1668.0 and p<0.0001). The post hoc Conover–Inman pairwise comparisons found that 5, 6 and 7 components formed a group with the highest accuracies. Other non-significant differences were found, but are not presented in detail, as they were cases with lower accuracies.

For the tests of the effect of epoch size, ANOVA and Tukey’s test results showed that while there was a significant effect of epoch size (F(ν=5)=4.3 and *p* < 0.001), only the 50 ms-long epoch size was significantly different from the others (*p* < 0.05, lower accuracy than the others).

**Figure 7:**
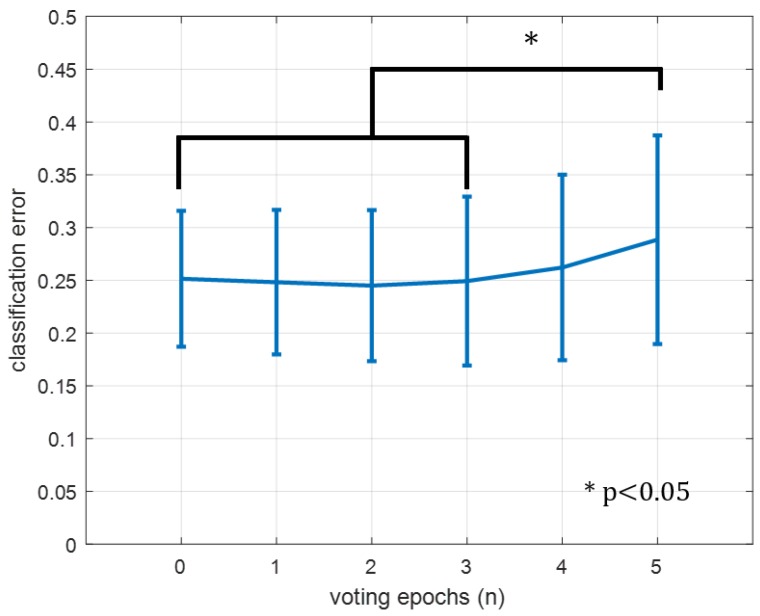
Effect of varying voting epochs on classification error. Zero epochs is equivalent to not voting and just accepting each epoch’s classification. Error bars are the standard deviation of the mean. * denotes significance at p<0.05.

When varying the number of voting epochs (Figure 7), there was no significant difference between using zero and four previous epochs in a unanimous voting scheme. There was a significant difference (p<0.05) between n=5 and n= 0–3.

### 3.2. Missed Transitions

Table 2 shows the normalized confusion matrices for the four classification methods. While the steady state is classified with 84%–87% accuracy, accuracy decreases to 68%–73% for grasp, and release is further decreased to 41%–49%. A similar trend is shown in Table 3, which breaks the accuracy for a single classifier down into precision, recall and F-score. Here, steady and grasp have higher precision than recall, which means that they have more false negatives, but fewer false positives. On the other hand, release has a higher recall than precision, the latter of which is less than 50%, so while it is classifying release hidden states correctly approximately 70% of the time, non-release states are also being labeled as such at a relatively high rate.

**Figure 8:**
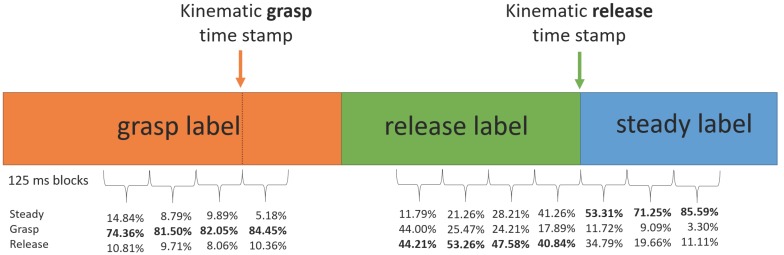
Enlarged version of the timeline from Figure 3, showing how often an epoch was classified as a given label for non-overlapping 125 ms windows close to kinematic time stamps for all trials in a five-component HMM unigram case. Bold numbers indicate correctly-classified regions.

Figure 8 shows the results of the predictions during the labeled regions surrounding the kinematic grasp and release time stamps (see Figure 3). We see that grasp is predicted correctly approximately 80% of the time during these periods both before and after the kinematic time stamp, while release is less likely to be correctly classified. The three blocks of 125 ms after the release time stamp show that steady and release are the two most likely labels, though steady (the correct label) is gradually predicted more as we move further from the release time stamp.

**Figure 9:**
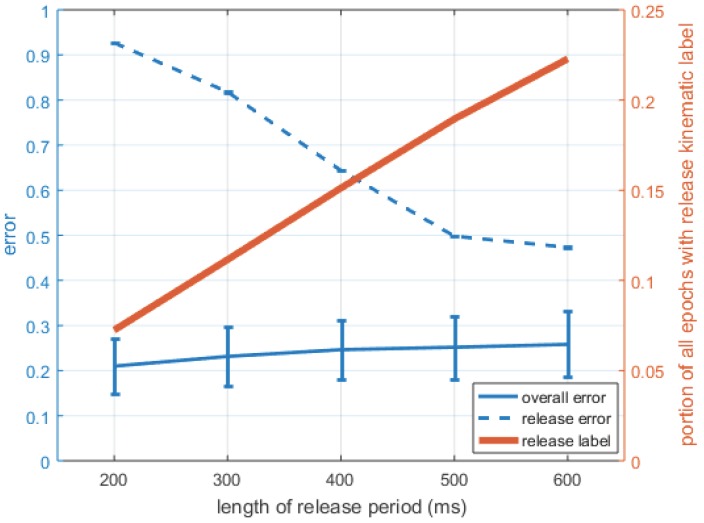
Effect of changing the length for which a kinematic label (hidden state) of “release” was applied for a five-component HMM unigram. Effects on classification error are shown for the overall accuracy of all states and specifically for release. The effect of changing the label length on the portion of all epochs that are labeled as release is also shown. Error bars are the standard deviation of the mean; error bars for release are present, but very small.

Figure 9 shows the effect of changing the length of time for which a kinematic label of “release” was applied. The error of release classification decreases as the length of the time window is increased, but the overall error of the entire trial increases.

## 4. Discussion

This study evaluated if pattern-recognition-based methods could classify transient, anticipatory signals. It also characterized the sensitivity of the top classifier to how the training data were labeled and to the use of a voting scheme.

### 4.1. Classifier Selection

To select a classifier, we evaluated if there were differences in accuracy between (1) HMM and GMM, (2) bigram and unigram and (3) different numbers of mixture model components. The data supported a significant difference for all three null hypotheses.

Post hoc tests showed that the unigram HMM generally performed better than the other classifiers. It was somewhat surprising that bigram HMM performed the worst of the four classifiers, though that may be due to overfitting to the training data, a particularly notable issue given that the training data consisted of about 90 grasp and release motions per person (cross-validation over a set of approximately 100 motions), a relatively small dataset for machine learning. We also observed that the overall shape of the test error curve in Figure 5 was characteristic of the typical model complexity/error plot for machine learning algorithms [35]. The near-zero slope and non-significant differences between the five- to seven-component cases suggests that a five-component model is likely the best choice, since it provides similar accuracy for less run-time.

The remaining classification error may be due to a number of different factors, including shifting sensor locations, pre-shaping and label source. As previously described, the data used in this study were pooled from trials involving five different sensor locations involving approximately 1-cm shifts on the forearm. Though we believe that shifts of these magnitudes will be common in typical usage of exoskeleton and teleoperation applications outside of the laboratory, they nonetheless add variability [24] and may contribute to the reduced accuracy. Both Gazzoni et al. [26] and Beckers et al. [24] show that sensor placement shifts like the ones in this dataset (which is the same as the Beckers et al. dataset) cause significant differences in the feature values, which would make the performance worse than if the classifiers had been trained on unshifted inputs. However, a direct comparison between the pooled and unpooled data was not possible using the current dataset due to the amount of data required. Further, collecting a dataset that would permit the exploration of pooled vs. unpooled conditions would require a larger dataset that may introduce other confounds, such as fatigue [36]. While this limits the ability to determine a baseline accuracy without varying sensor placement, it does not affect the ability to further examine sensitivity to label lengths and voting schemes.

Pre-shaping motions may also have contributed to the reduced accuracy during transition periods, as these motions have muscle activations that are similar to a full grasp-release sequence [23,25,37], but are still kinematically labeled as steady epochs. A likely example of this can be seen in Figure 4, around Epoch 160, which is similar to many other such occurrences in other trials. Due to the differing nature of the sEMG and kinematic labels, pre-shaping might be detected by the classification algorithms, but remains labeled as steady-state in the kinematic labels because such smaller actions were not labeled and trained in the dataset. Pre-shaping presents challenges to learning algorithms for two reasons. First, it confuses the training since it adds signals related to the shaping component that is related to grasping, causing steady to be labeled as grasp, which we see 13%–22% of the time. Second, it affects our definition of accuracy since the signal may be more indicative of grasp with pre-shaping, but our labeling does not permit a pre-shaping state.

Many of the classification errors occurred during epochs of state transitions, where there was a lead or lag in the state labeling (e.g., Figure 4, Epochs 50–100). This phenomenon was also noted by Chan and Englehart [13] in their HMM gesture classification system. Chan and Englehart [13] overcame this problem by removing HMM outputs within 256 ms of a transition period when conducting their accuracy calculations, yielding an accuracy difference of 0.5% compared with classification accuracy measurements obtained without the removal of these outputs. However, they did not consider the inclusion of such outputs to be a significant issue when implemented on a device controller, since they were interested in static gestures, and the inertia of the actuator’s movement would likely overcome short periods of misclassification. On the other hand, since we are specifically interested in dynamic transitions as a method of controlling an exoskeleton device, removing such periods from our analysis would not be appropriate.

The analysis of the epochs around the kinematic grasp and release time stamps revealed that grasp was well-predicted at those times (Figure 8, left), while release was less so (Figure 8, middle), matching with the overall results in the confusion matrix (Table 2). The precision/recall table (Table 3) further describes these data, highlighting that release tends to have more false positives (i.e., the label was applied more often to the wrong hidden state) than false negatives (i.e., the label was not applied to an actual release hidden state). For periods after release (Figure 8, right), there is initially some confusion over whether the intention is steady or release, but the correct prediction of steady becomes increasingly likely when moving away from the release period. Since greater force is required to hold the cup than to release it, activation of the flexors in the grasp phase may generate a more detectable signal. In contrast, release requires activation of the extensors, but a gradually-decreasing applied force, so the signal there is less detectable. Further, after the kinematically-labeled release, the fingers are still extending, thus activating the extensors within the initial part of the steady region. Additional data inputs (e.g., more sEMG sensors or other types of data) may help reduce these false negatives and false positives.

### 4.2. Label Length and Voting Scheme Analyses

From the classifier evaluation, the five-component unigram HMM was selected for further analysis. In varying the length of the kinematic release labels (Figure 9), we find that longer release labels result in lower release classification error, but conversely, in higher overall classification errors when considering all epochs. When the length of the release period is short, the portion of epochs labeled as release is correspondingly small. This provides fewer release-labeled data points on which the HMM can train. With longer release periods, there was a larger training set of release-labeled data. However, after some threshold (which appears to be the “elbow” at 500 ms), the benefits of the larger training set are likely diminished by over-smoothing from the larger label region, as more of the window was further from the kinematic time point of release.

This analysis provides a specific guideline for implementing sEMG-based pattern-recognition controllers that was not explored in previous literature. Operational use cases with dynamic motions are typically composed of a sequence of several sub-movements of different lengths. Recognition of shorter sub-movements requires more training trials than recognition of longer sub-movements. Here, grasp was more likely to be classified correctly, in part since there were more grasp-labeled data than release-labeled data (Figure 3 and Figure 9). If a release label length should be shorter than the the grasp label length (which is likely given the differing force and muscle activation requirements for each), then more data may be needed to lower that specific sub-movement’s classification error. The shorter the shortest sub-movement is in a sequence, particularly in comparison to other sub-movements, the more overall training is required to correctly detect it. Thus, training for natural dynamic actions requires greater consideration for not only the types of actions being performed, but also for how long they are performed. This is a factor not considered in previous literature, where different gestures were held for equal periods of time [13,14].

Another guideline provided by our analysis was that voting schemes may be useful for increasing classification accuracy through the removal of spurious classifications, but use of too many epochs can degrade performance by over-smoothing. Although epoch-based voting was previously used in an HMM context by Chan and Englehart [13], our procedure specifically examined this as a variable and only used information preceding the decision point, showing the results of voting in an online application, rather than for post hoc analysis.

### 4.3. Comparison to Literature and Additional Limitations

Overall, the best classification accuracies we achieved for transient anticipatory detection were lower than those of sEMG-based static gesture classification studies (e.g., [13,14]), which were typically able to achieve classification accuracies around 90–95%, though in the case of Wolf et al., this was accomplished with additional information from IMU data. There are a number of differences in these studies that may account for the lower accuracy here. First, our subjects had a high degree of freedom with regard to how they performed the grasping task, as the experiment directions did not specify a particular way to grasp, nor did they specify speed or accuracy objectives, both of which affect grasp size and, consequently, sEMG signals [38]. Second, the actions we were classifying were dynamic rather than static gestures. Third, our participants performed contact gestures where they were touching an external object (the cup) part of the time. Collins [39] notes that object contact causes a significant change in the sEMG signal from the activated muscles, which also depends on where the contact actually occurred. This third factor also interacts with the confounding factor of our participants likely touching the object in different ways during different trials, which may generate different co-contraction responses and, thus, add variability to the underlying actions that were used for training and testing. As previously mentioned, we also pooled data collected under different sensor band configurations, while previous studies did not have this additional confound. Finally, with specific reference to the HMM-based studies by Chan and Englehart [13] and Marchal-Crespo et al. [22], we used the forwards algorithm rather than the forwards-backwards algorithm, which would have smoothed out our posterior state probabilities, but made the technique a solely post hoc rather than a potentially online method. These limitations highlight the complexity of using these algorithms directly in a natural task environment. However, the conditions under which our data were collected are likely more representative of the practical conditions under which an assistive or augmentative exoskeleton would be used.

It should be noted that since we used a direct epoch-by-epoch comparison, some losses in accuracy will likely contribute little to any increase in difficulty with using this method as a controller. A lead or lag time on the order of tens of milliseconds is generally acceptable for exoskeleton and prosthetic control [40]. A brief “twitch” in the exoskeleton’s actuation, potentially caused by the grasp-release sequence extracted from a pre-shaping motion, may not have a major negative effect. It is possible that such a “twitch” could be a vibrotactile cue and could augment visual cues to ensure that an exoskeleton or prosthetic user knows what the device is about to do and learns to adapt to it more easily [41,42].

The levels of accuracy achieved here still exceeded the 70% accuracy value recommended for device control with brain-computer interfaces [40] as an overall average and for steady and grasp states individually, though not for the release state by itself. Further algorithmic work is warranted to increase the accuracy to usable levels when integrated with a human in the loop. Specifically, a number of the sEMG feature parameters (such as the Wilson amplitude threshold, the frequency bin edges and amplitude bin edges) were not manipulated in this experiment, and better results may be possible under different parameter settings than those that were used here. Alternatively, hardware changes, such as the use of other kinds of data (e.g., position or velocity), a change in sEMG placement or additional sEMG sensors (as in Wolf et al. [14]), could also contribute substantially to improvements in detection accuracy. This study examined a single object of a single weight, which limits interpreting the generalizability of the method. Previous work [43] indicated that EMG patterns of grasping show distributions that are independent of the force exerted by the grasping digits, which may indicate that pattern recognition methods like the ones used here would still apply across different object weights. Future work on testing these methods with objects that require different types of grasps or objects of different weights are certainly still warranted for further direct validation.

### 4.4. Implications for Use with Exoskeletons

One of the key differences between the data used in this study and the conditions that would be encountered during actual exoskeleton use is that participants in this study did not receive assistance from any external device when coming into contact with the objects they were transporting. Given the differences between the sEMG signals of people performing grasping actions with and without physical contact with an object [39], it is assumed that additional contact with an exoskeleton device would further alter the sEMG signals during a grasp-and-release task. Thus, the data in this study are insufficient to demonstrate the direct applicability of the described methods to controlling an sEMG-based exoskeleton device.

However, the overall data processing and training/testing pipeline developed in this study is not specific to the conditions under which the input signals were collected, as long as some action labeling is provided for training. Indeed, it is not even specific to sEMG signals, so other sensor information, could also be included. It is also hypothesized that users of an sEMG-based controller may learn muscle activation patterns, such as exaggerated movements or co-contractions, which increase the exoskeleton’s ability to detect their intentions over time. Future work on the controller side may also look into how the classification algorithm could adapt to the user over time, as well, allowing for co-adaptation in the human-machine system.

## 5. Conclusions

We provide contributions in two areas: (1) testing different models for classifying transient sEMG signals in grasp-and-release motions under a protocol that mimics an operational use case and (2) expanding the understanding of how classification accuracy is affected by the choice of labeling periods during training and the use of voting schemes. Our experiment protocol classified sEMG signals from transient contact motions with shifted, non-muscle-specific sensor placement. This stands in contrast to previous studies of sEMG gesture classification [13,14,17], where static gestures without object contact were used and sensor placement was muscle-specific [17] and/or held constant [13,14,17]. The use of non-specific and shifted sensor placement allowed us to incorporate a source of noise that is likely to occur in operational use cases.

This study expanded the understanding of HMM implementations in sEMG classification settings by considering the sensitivity to a number of components, the type of data, label lengths and voting epochs. In doing so, we were able to provide specific implementation guidelines for training and using HMMs in an online control context. Furthermore, in contrast to previous literature [13,21], we considered an online implementation of HMM inputs by using only the forward algorithm for classification, avoiding the typical forwards-backwards algorithm and past/future voting, which utilize future information that would only be available post hoc.

A specific implementation guideline provided by our analysis was related to the relative lengths of sub-movements to developing protocols for training operational systems. Since movements comprised of sections of different lengths are likely to be trained together in a set, the length of the shortest sub-movement presents a limitation in classification accuracy, since the least training data will be collected on it. This limitation must be considered when selecting training sets for pattern recognition models to recognize sets of movements, especially when used for dynamic motions. The basic framework of data processing, training and testing developed here is sufficiently flexible to analyze the results of such experiments and also be applied to a controller with different sets of inputs apart from sEMG, without loss of generality.

## Figures and Tables

**Table 1:** Subject characteristics.

Characteristic	Min	Max	Mean	Standard Deviation
Age (years)	19	30	23.6	3.13
Forearm Circumference * (cm)	22.0	28.5	25.7	1.91
Forearm Length ** (cm)	19.5	29.6	24.8	2.42
Seated Shoulder Height (cm)	53.0	63.0	58.3	3.16
Mass (kg)	59.9	83.9	75.2	7.19

* Measured at 25% the length of the ulna as measured from the lateral epicondyle; ** measured from the lateral wrist to the lateral epicondyle.

**Table 2:** Normalized confusion matrices for the four classification methods when using 5 mixture components. Rows are ground truth (kinematic) labels; columns are predicted labels; and S, G and R correspond to steady, grasp and release labels. All values are percents, and values along the main diagonal are correct predictions. Steady states occur most often; this means that the subject is not in any of the other states.

	**(a) Unigram GMM**						**(b) Bigram GMM**			
		**Predictions**						**Predictions**		
		S	G	R				S	G	R
labels	S	84.6869	7.3709	7.9422		labels	S	85.4543	7.2222	6.3235
	G	13.5847	72.6140	13.8013			G	16.2129	72.6117	11.1754
	R	23.0644	27.6310	49.3046			R	25.7886	30.6402	43.5711
	**(c) Unigram HMM**						**(d) Bigram HMM**			
		**Predictions**						**Predictions**		
		S	G	R				S	G	R
labels	S	86.3376	6.1729	7.4895		labels	S	87.2922	6.8754	5.8324
	G	15.3602	72.9194	11.7204			G	21.9088	68.0618	10.0294
	R	23.5518	28.8595	47.5888			R	30.8624	28.3947	40.7429

**Table 3:** Accuracy measures for a 5-component unigram HMM. All values are percents.

	Steady	Grasp	Release
precision	86.34	72.92	47.59
recall	68.93	67.55	71.24
F-score	76.66	70.13	57.06
